# Exploiting Polyhydroxyalkanoates for Biomedical Applications

**DOI:** 10.3390/polym15081937

**Published:** 2023-04-19

**Authors:** Vipin Chandra Kalia, Sanjay K. S. Patel, Jung-Kul Lee

**Affiliations:** Department of Chemical Engineering, Konkuk University, 120 Neungdong-ro, Gwangjin-gu, Seoul 05029, Republic of Korea; vckaliaku@gmail.com (V.C.K.); sanjaykspatel@gmail.com (S.K.S.P.)

**Keywords:** polyhydroxyalkanoates, biopolymers, biomedical, microspheres, drug delivery, wound healing, tissue engineering, biocontrol

## Abstract

Polyhydroxyalkanoates (PHA) are biodegradable plastic. Numerous bacteria produce PHAs under environmental stress conditions, such as excess carbon-rich organic matter and limitations of other nutritional elements such as potassium, magnesium, oxygen, phosphorus, and nitrogen. In addition to having physicochemical properties similar to fossil-fuel-based plastics, PHAs have unique features that make them ideal for medical devices, such as easy sterilization without damaging the material itself and easy dissolution following use. PHAs can replace traditional plastic materials used in the biomedical sector. PHAs can be used in a variety of biomedical applications, including medical devices, implants, drug delivery devices, wound dressings, artificial ligaments and tendons, and bone grafts. Unlike plastics, PHAs are not manufactured from petroleum products or fossil fuels and are, therefore, environment-friendly. In this review, a recent overview of applications of PHAs with special emphasis on biomedical sectors, including drug delivery, wound healing, tissue engineering, and biocontrols, are discussed.

## 1. Introduction

Polyhydroxyalkanoates (PHA) are a class of biodegradable polymer-based materials that have garnered substantial attention for their potential medical applications [1,2,3]. These polymers contain carbon and hydrogen, with ester linkages between the hydroxyl groups. Microorganisms produce esters via fermentation; the microbes consume sugar and convert it to fat for storage and energy production. These polymers are produced via fatty acid synthesis, and the enzymes involved are synthases. Unlike plastics, PHAs are environmentally friendly because they are not made from petroleum products or fossil fuels. Bioplastics have several advantages over conventional plastics, such as a low carbon footprint, energy efficiency, versatility, unique mechanical and thermal characteristics, and societal acceptance. Bioplastics are unique due to their biodegradability; moreover, they can be sterilized without causing damage to the material itself. These properties render PHA ideal for medical devices and implants [4]. In addition to being biocompatible and biodegradable, PHAs are inexpensive to produce compared with other bioplastics; therefore, its large-scale production is cost-effective [5]. The PHA extraction process depends upon the bacterial culture and physico-chemical properties of the polymer (molecular weight, polydispersity index). The main recovery approaches involve solvents (alcohols, alkanes, halogenated solvents, carbonates, esters, and ketones) and cell lysis (enzymes, oxidants, surfactants, acid, and alkaline compounds) [6]. Globally, the PHA market was valued at approximately USD 73.6 million in 2021 and is projected to grow to USD 167 million by 2027 [7,8]. Bioplastics with potential biomedical applications include PHA, polylactic acid, poly-3-hydroxybutyrate (PHB), biopolymers (based on cellulose, lipids, proteins, and starch), polyamide 11, and polyhydroxyurethanes [9]. Among the various applications of PHAs, tissue engineering, which includes restoring or replacing damaged organs or tissues, was expected to grow from USD 9.9 billion at a rate of 14.2% between 2019 and 2027 [10,11]. The status of bioplastic production capacities (~103 tons) varies by sector: (i) coatings and adhesives (35.2), (ii) electrics and electronics (~10), (iii) agriculture and horticulture (~90), (iv) consumer goods (~200), (v) fibers (~30), (vi) rigid packaging (~200), and (vii) flexible packaging (~400), and to a minor extent, for articles such as food, coffee pods, compostable cutlery, edible films, bags, biomedical tools, and bottles [12,13,14].

Several types of PHAs with different structures and properties can be used for various purposes. For example, some types are bioerodible, which means that they completely break down in the body over time, while others persist for longer. The different properties of each type of plastic make them more suitable for certain applications [15]. For example, copolymers of PHAs are mechanically stronger than homopolymers. Consequently, they are useful for devices, such as surgical sutures, that need to withstand considerable stress without tearing. PHB is a highly crystalline microbial polyester that belongs to the PHA family. It is currently being tested as a controlled-release implant for drug delivery to treat cancer and other chronic diseases. PHAs have many potential applications in biomedical engineering [16]. Recently, these plastics have been investigated more thoroughly, and new applications are constantly being developed. Because they do not contain toxic substances, they can be safely used in the human body without fear of adverse side effects. In addition, most types of PHAs can be synthesized from pure sugars such as xylose and hexanoates [17] and renewable resources such as corn, and many biowastes of plant and animal origin can be used as feed for the microbial production of PHAs, which makes it environmentally friendly [18,19,20,21,22,23]. However, PHAs have several disadvantages. Some are not suitable for use with biological tissues because they are water-soluble and not very stiff. In addition, the manufacturing process is expensive and requires specialized equipment. Therefore, further investigation is required before they become more cost-effective and applicable than plastics for medical applications. In fact, PHAs must be modified to produce scaffolds for biomedical applications [24].

Biodegradable and porous scaffolds have been manufactured via various techniques: (i) electrospinning—from (a) poly3-hydroxybutyrate-co-4-hydroxybutyrate (P34HB)—fiber scaffold, nanofiber membrane, (b) PHB—microfibers and nanofibers, nanotubes scaffolds, nanofiber scaffold, conduit, (c) PHB and poly(3-hydroxybutyrate-co-3-valerate (PHBV)—fiber, (d) PHBV—membrane, nanofibers, tissue-engineered vascular graft, patches, fibrous scaffold, nanofiber film, fibrous scaffolds, nanofibrous scaffold, composite nanofiber, hydrogel patches, nanofibrous mat, (ii) co-precipitation from PHB—implant, (iii) Salt leaching technique and 3D- printing from PHB—Bioactive biopolymer/mineral/hydrogel scaffold, bone grafts, (iv) solution casting from poly(3-hydroxybutyrate-co-3-hydroxyhexanoate (PHBHHx)—film, (v) solvent evaporation from P34HB—film, (vi) solvent casting- particulate leaching from PHBHHx—porous structure scaffold, (vii) solution casting from PHBV—film [25,26,27,28,29,30,31,32,33,34,35]. This article aims to provide a comprehensive overview of PHA as an alternative to synthetic plastics in advanced biotechnological applications, including drug delivery, wound healing, tissue engineering, and as biocontrol agents.

## 2. Biopolymer-Synthetic, Biopolymer-Inorganic Composites

The characteristics of various PHA devices can be engineered by producing their blends and composites. Desired mechanical strength, degradation rate, and biocompatibility can be achieved for successful tissue engineering and drug delivery [26,36]. A few examples of PHAs and their composite-based scaffolds for biomedical applications are as follows: (i) PHB/ZrO2/Herafill^®^ prepared through Injection-moulded cylindrical pins resulted in enhancing bone growth on 30% HerafillR composite [37], (ii) PHBV microspheres coated 45S5 bioactive glass-based construct via foam replication and dip coating 3D scaffold had the following characteristics—well-regulated drug delivery and a significantly improved compressive mechanical stability of scaffold [38], (iii) PHBHHx)/Mesoporous 45S5 Bioglass^®^ fabricated using 3D printing technology had a few unique features such as hierarchical pore architecture, improved strength, higher bioactivity and significantly higher bone formation [39], (iv) nano-HA were incorporated into a P(3HB) matrix, which showed significantly higher cell proliferation and differentiation [40], (v) PHBV)/β-Ca_2_SiO_4_ fabricated using solvent casting, and two size salt particles, particulates leaching resulted in a composite with highly interconnected pores, enhanced hydrophilicity, higher cell proliferation/differentiation [41], and (vi) cardiac patches based on P(3HO) had mechanical strength equivalent to that observed for cardiac muscle. High biocompatibility with neonatal ventricular rat myocytes also had cell viability, proliferation and adhesion on (P(3HO) films [28].

## 3. Biomedical Applications

**Figure 1:**
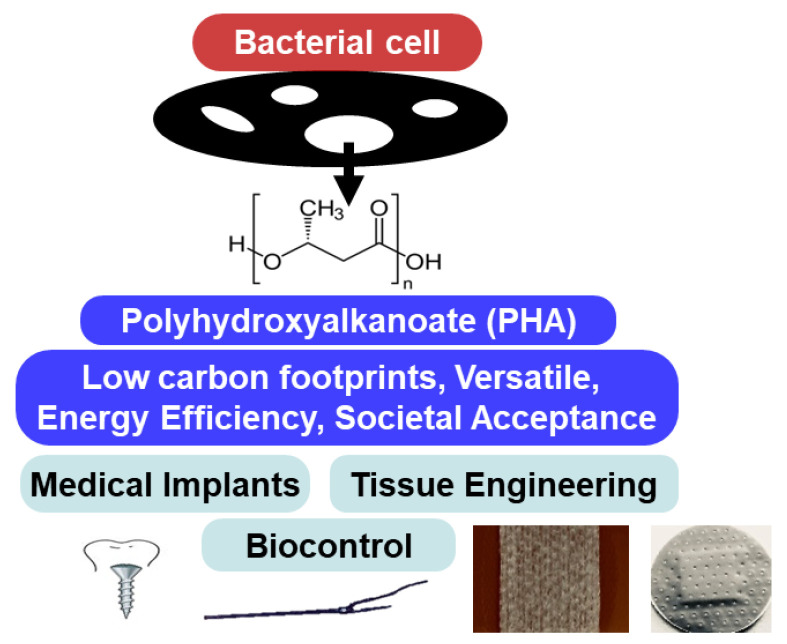
Biomedical applications of polyhydroxyalkanoates.

Many natural polymers, such as collagen and fibrin, are biocompatible but lack the necessary biodegradability required for successful medical applications. Hence, developing novel synthetic materials suitable for both medical and industrial applications has been of interest. Applications of PHAs for biotechnological purposes have been exploited in diverse fields, ranging from aquaculture to human health. Their economic value is relatively high because of their biodegradability, biocompatibility, and non-toxic nature. Recently, the use of PHAs has become an essential area of research because of their potential use in producing functional and biodegradable materials for a wide variety of biomedical applications, including tissue engineering (cardiac- and coronary-related bone reconstruction), drug carriers and delivery, medical implants, and biocontrol agents (Figure 1) [15,16,42,43,44].

### 3.1. Tissue Engineering

PHAs with varying structures and properties can be used to produce scaffolds for regenerative medicine [35]. Ultra-high-purity PHAs are required for tissue engineering applications. Biodegradable materials are essential because they minimize the risk of infection after implantation and enable the body to naturally heal the injured site sans surgical intervention [45]. However, the use of synthetic or non-biodegradable materials may result in complications such as scar tissue formation and subsequent lack of healing. Hence, PHA-based materials must be evaluated under in vivo conditions and, if necessary, modified to show the requisite properties for fabricating scaffolds and ensure time-bound biodegradation. Such well-engineered PHAs can be used to develop tissue-based products, achieve efficient tissue engineering, and meet the therapeutic needs of nerve tissues, heart valves, stents, and vascular grafts [25,26,27]. PHA modifications can help improve the mechanical strength and produce scaffolds that promote cell growth [46]. These properties can extend the range of biotechnological applications of PHAs to produce sutures, films, pins, and screws [47]. PHA copolymers based on multiple monomeric types of poly(3-hydroxybutyrate-co-4-hydroxyvalerate-co-3-hydroxyhexanoate) [P(3HB-4HB-3HV)] have been used for fabricating fibrous meshes, which can support the growth of stem cells [48]. Copolymeric PHA scaffolds have specific applications depending on their monomeric components: (i) poly(3-hydroxybutyrate-co-3-hydroxyvalerate-co-3-hydroxyhexanoate) [P(3HB-3HV-3HHx)] for liver tissues [49], (ii) 3-D scaffolds for nanofibers [50], and (iii) poly(3-hydroxybutyrate-co-polyhydroxyoctanoate) [P(3HB-3HO)] for cartilage repair [51]. PHAs combined with inorganic bioceramics have high flexibility and mechanical strength, and they can be easily blended, making them suitable for producing novel composites such as hydroxyapatite for engineering tissues [52,53]. To improve the efficiency of sutures, autodissolution after a certain period is highly desirable. Using a copolymer poly(3-hydroxybutyrate-co-3-hydroxyhexanoate) P(3HB-3HHx)-based suture resulted in 58.5% weight loss over seven weeks [54].

A few PHB-based items, such as non-woven patches and porous scaffolds (mesh-like structure), have been observed to assist in regenerating organs ranging from osseous, cardiac, intestinal, neural, and vascular tissues. The materials were found to induce vascularization in defective regions [55,56]. In contrast to homopolymers such as PHB, PHA copolymers including PHBV, poly(3-hydroxybutyrate-co-3-hydroxyhexanoate) (PHBHx), and poly(3-hydroxybutyrate-co-3-hydroxyhexanoate) (PHB-VHx) have been proven to be instrumental in stimulating the proliferation of HaCaT human keratinocytes [57]. Nanoparticles (NPs) of PHB-VHx (0.02–0.1 g/L) enhanced cell proliferation by activating cell division. This enhancement was due to an increase in the concentration of cytoplasmic calcium ions. Interestingly, the PHA metabolic degradation product 3-hydroxybutyric acid (3HB) (0.1–1.0 mM) also effectively activated cell division in L929 murine fibroblasts and *HaCaT* human keratinocytes. Moreover, fibroblast apoptosis and necrosis were suppressed [58,59]. The proliferation of pancreatic beta cells in mice and suppression of apoptosis, without any cytotoxicity, was observed in a wide range of homopolymers (3HB), PHA oligomers, and copolymers consisting of 4-hydroxybutyric acid and 3HB (20 µg/mL) [60]. A copolymer consisting of poly(3-hydroxybutyrate-co-3-hydroxyvalerate-co-2,3-dihydroxybutyrate), produced by *Cupriavidus eutrophus* was found to be effective in fabricating films suitable for regenerating soft tissues [61,62]. Scaffolds with low cytotoxicity have been used to enhance the osteogenic differentiation of osteoblasts and mesenchymal stem cells (MSCs) in humans and animals such as rats and rabbits [63,64,65,66,67]. This growth and differentiation are regulated by the topography and microstructure of the devices [68]. The PHA degradation product 3HB (>0.05 mM) promoted the osteogenic differentiation of osteoblasts [69]. Copolymers-PHBHx stimulates chondrogenic/neurogenic differentiation of MSCs by influencing the expression of the following genes: (i) *sox9*, *pthrp*, *col2*, and *col10* [70], and (ii) encoding nestin, acidic protein, glial fibrillary, and βIII-tubulin [71,72]. Monomers of PHAs, 3HB, act as neuroprotective agents by providing energy to neurons and stimulating signal transduction. These metabolic processes are mandatory for enhancing memory and learning [73]. In certain cases, implantation is ineffective in regenerating defective bone tissues. In rat femur and skull models, the expression of type I collagen was determined as an osteogenic marker that regulated the bio-resorption of polymeric material and associated processes, such as vascularization and intergrowth into PHA scaffolds [28,56]. Tissue engineering scaffolds with myoblast cell lines showed very high biocompatibility when PHA copolymers, poly(3-hydroxyhexanoate-co-3-hydroxyoctanoate-co-3-hydroxydecanoate-co-3-hydroxydodecanoate) were produced from biodiesel industry waste as feed. The net gain was 72% higher than that of the C2C12 cell line [74]. Scaffolds with MSCs caused a 3.5-fold higher regeneration of bone defects in rats within a short span of 3–4 weeks [31]. The adhesion and lifespan of fibroblasts and neuronal cells were substantially improved when PHA-copolymer-based composites, films, and microfibers were used. Microbes, such as *E. coli*, *Parabrkholderia*, and *Pseudomonas,* are among the most effective PHA producers [75,76,77]. The main disadvantage of using PHAs for tissue engineering applications is their poor mechanical strength, which limits their use in load-bearing applications. Moreover, leaching bioactive molecules from scaffolds can lead to cytotoxic effects at the implantation sites. However, this limitation has been overcome by cross-linking poly[(R)-3-hydroxybutyrate-co-(R)-3-hydroxy-10-undecenoate] via thiol-one click chemistry. The cross-linked polyester had an increased tensile strength with physical characteristics relevant to soft-tissue replacement and did not exhibit any significant cytotoxicity [31]. Recent studies in tissue engineering further support the low or non-cytotoxicity of scaffolds [65,66,67]. Commercially produced PHB and PHA copolymers have enabled improvements in skin generation during wound healing, resulting in better cellular responsiveness in diabetic models and reducing unnecessary scar formation [29,30,32,33,34]. The diversity of biomedical applications of PHAs in tissue engineering has been presented in Table 1.

The synthetic polymer poly(glycerol sebacate) was modified by 3D-printing technology using PHB and nano-HA to produce scaffolds useful for reconstructing the craniofacial bone [78,79]. PHA copolymer [PHBHHx (2 mol%)] produced on a large scale using a genetically modified *Cupriavidus necator* strain had unique characteristics (low melting point and glass transition temperatures) suitable for skin tissue engineering [80].

Tissue engineering scaffolds provide 3-dimensional support during tissue repair especially cell attachment and maturation [81,82]. In addition, functional scaffolding biomaterials such as bioactive glasses, nanocomposites of hydroxyapatite, and electrically conducting hydrogels play a vital role in regulating cell behavior. A few other features of importance in regenerative medicine are the antimicrobial surface coatings for biomedical implants and scaffolds and bioactive molecule-releasing scaffolds [83]. The biomolecule-releasing ability of the biocomposite scaffolds is desirable for improving bone regeneration efficiency. 3D nanofibrous scaffolds with the well-regulated release of growth factor (BMP-7) were fabricated by encapsulating them into the polymer microsphere. These were then immobilized on nanofibrous scaffolds. The controlled release of growth factors resulted in high ectopic bone formation [84]. Antimicrobials releasing scaffolds are designed to inhibit microbial colonization on implant sites. Poly(d, l-lactic acid) nanofibrous scaffolds treated with Silvadur ET released silver ions, which restricted bacterial growth [85]. Another feature important for tissue engineering and drug delivery is the stimuli-responsive materials [86]. These polymers can self-assemble or undergo morphology transformation or phase transitions. Piezoelectric scaffolds, which can cause electrical stimulation on cell growth, differentiation, and tissue growth, have potential applications in tissue repair and bone regeneration [87,88,89,90].

The structural features of scaffolds are critical in tissue engineering employed for the restoration and maintenance of injured tissues and organs [91,92]. Various fabrication techniques such as 3-D printing, etching, electrospinning, magnetic, and freeze-casting enable achieving scaffolds with varied topographic orientations. These features influence the efficiency of the regeneration of tissues and organs. The underlying mechanism in aligned and random orientation influences the biological responses in cells. Recent efforts are targeted to develop biomimetic scaffolds which stimulate the structure and composition of the extracellular matrix of the native tissues [93,94].

### 3.2. Drug Carriers and Delivery

Among the various approaches attempted to improve drug efficacy, well-regulated delivery has been found to be critical. Owing to their flexibility, durability, biodegradability, and biocompatibility, PHAs have been targeted as feed for producing NPs, and as scaffolds for eluting drugs [95,96]. Their use for controlled and sustained drug delivery to wounds improves the efficiency of therapeutic molecules with minimal side effects. For example, silicone, which has been frequently used to encapsulate hydrophobic drugs, must be replaced as it is carcinogenic [97,98]. Dendrimers are produced from 3HB monomers. These biopolymers have unique features, such as monodispersity and functional moieties on the surface, which promote their use as drug carriers [99,100]. Novel b- and c-peptides resistant to peptidases can be prepared from 3HB and 4-hydoxybutyric acid, which can be sustained for a long period within the mammalian serum by ensuring well-regulated drug delivery. The PHA monomer 3HB can be utilized for producing fragrance (S-citronellol) and sex hormones [101]. PHA copolymers—PHBHV)-and 3-hydroxybutyrate-co-4-hydroxybutyrate [P(3HB-4HB)]- based rods were used as implants that could release antibiotics. Microspheres fabricated from PHB were used to effectively transport and release hemoembolizing agents (rifamycin) on-site in a sustained manner [102]. The physicochemical properties of mcl-PHAs, such as their low crystallinity and melting point, proved effective for transdermal drug delivery. PHA-copolymers composed of monomers 3-hydroxy octanoic acid and 3HHx could adhere efficiently to python reticulatus skin. This enabled the easy dispersal of drugs, including clonidine, ketoprofen, and tamsulosin, owing to their enhanced permeability through the PHA matrix [103]. mcl-PHAs obtained from *Pseudomonas fluorescens* have multiple applications in diagnostic tools, such as drug delivery, immobilizing agents, and protein purification [104]. Modified PHAs as biologically active beads have been demonstrated to help in developing skin test reagents, diagnosis, production of recombinant proteins, delivery of vaccines, and removal of endotoxins [105,106,107,108,109,110,111,112,113,114,115,116].

Using PHA copolymer, P(3HB-HV)-based microspheres mixed with a surface stabilizer (polyvinyl alcohol) substantially improved tetracycline loading and a well-regulated release of the antibiotic used for treating periodontal infection caused by *Porphyromonas gingivalis* and *Actinobacillus actinomycetemcomitans* [117]. Loading antimicrobial compounds such as epirubicin, doxorubicin, or curcumin on PHA copolymers could enhance cell viability, which was accompanied by retardation in the growth of pathogenic bacteria [59,118,119,120]. Immobilization of lysozyme on electrospun sheets made up of P(3HB-30 mol% HHx) at the rate of 16.1 µg enzyme/9.5 mm^3^ discs could effectively inhibit 42% of the total biofilm formation by pathogenic bacteria. Biofilm inhibition was 12% higher than that achieved using solvent-cast sheets. Thus, wound dressings based on such sheets can more effectively eradicate bacterial infections [121].

Restricting cell proliferation is a strategy for cancer management. *Pseudomonas putida* CA-3 is known to produce medium-chain-length (mcl)-PHA, which has improved physicochemical properties compared to PHB. Depolymerized mcl-PHA generates monomers, largely (R)-3-hydroxy decanoic acid (R10), which, when conjugated with D-peptide DP18, showed a substantially enhanced anticancer proliferation activity. A 3.3–6.3 higher IC_50_ value was observed when conjugating peptides to hydroxylated decanoic acid (ω-hydroxy decanoic acid). The uptake of peptides into MiaPaCA and HeLa cells induced apoptosis [122]. PHA copolymers with a high hydroxy valerate content (P(3HB-3HV, 5–15 mol%)) obtained from *B. cereus* were used to produce drug nanocarriers. These nanocarriers were used to carry ellipticine (a plant alkaloid). The biomolecule content varied between 39–45%, which was influenced by the contribution of HV to the copolymer as PHA-polyvinyl alcohol (PHA-PVA). These anticancer agents are efficient because of the high biocompatibility of nanocarriers [123]. The delivery of anticancer drugs through these NPs based on PHA-polyethylene glycol (PHA-PEG) was efficient for a sustained response. The use of nanocarriers in cancer therapy has been demonstrated using cisplatin, which led to higher apoptotic activity in HT22 cells [124]. P(3HB)-based spherical polymeric nanocomposites with a higher anticancer drug (docetaxel) loading capacity (>17%) showed enhanced (>43%) encapsulation efficiency [125]. The information regarding the applications of PHAs in drug carriers and delivery has been presented in Table 2.

To develop the wound dressing necessary for healing defects in the skin of Wistar rats, the copolymer P(3HB-4HB) was used. PHA-based membranes loaded with fibroblasts from mesenchymal stem cells facilitated the release of fibroblast-secreted matrix proteins. These could facilitate the migration of epidermal cells to the wound site, which proved to be effective in enhancing the wound healing process by 1.4-fold compared to a cell-free membrane and 3.5-fold more rapid than control-eschar. This improved wound-healing process also showed reduced skin inflammation [126]. To repair damaged tissue rapidly, new cells should be able to proliferate vigorously. PHB scaffolds and surface-modified electrospun (laminin) fibrous material together enabled the cellular viability of the murine neuroblastoma Neuro2a cell line to increase from 116% at 4 h to 187% after 72 h of seeding [127].

### 3.3. Medical Implants

The human body has a well-developed immune system that reacts strongly to foreign bodies by secreting pro-inflammatory cytokines. However, PHAs are biological in origin and, therefore, biocompatible. This property enables their use in medical devices such as surgical sutures and dental implants. Because PHAs degrade in the body, their impact on long-term side effects is quite low. PHAs are suitable for applications in which a permanent device is not desirable. PHAs are advantageous for medical implants because they are biocompatible and biodegradable. Owing to their physicochemical properties, thermoplastic polyesters (PHAs) can also be used to produce customized shapes and sizes for specific applications [128,129]. Medical implants based on PHB and its copolymers have several advantages. Because of their high biocompatibility, strength, and slow degradation, biopolymers have been proven to be suitable for fabricating a wide range of resorbable medical devices. These devices range from surgical sutures, porous matrices, scaffolds, microspheres, woven mesh endoprostheses, orthopedic pins, meniscus repair devices, screws, staples, stents, stacks, rivets, surgical meshes, sutured fasteners, and plug endoprostheses to scaffolds for application in mammalian and human cells, including potent drugs (psychoactive neurotransmitters), regeneration of soft tissue during repair of hernia, and cancer cells [21,130,131].

Matrices based on P3HB and P3HO lead to the extensive production of drug-eluting coronary stents to prevent arterial blockage [132]. Biodegradable subcutaneous implants in rats were supplemented with 10-undecanoic acid and octanoic acid produced by *Pseudomonas oleovorans* [133,134]. Nanocomposites based on poly(3HB-co-70%4HB) from *Cupriavidus* strain and Claytone APA (5% *w*/*w*) exhibited highly improved physicochemical properties such as high strength (17 MPa Young’s modulus), low melting temperature (T_m_), and high transparency. These composites are highly suitable for producing green materials and regenerative medicine. The di-alkyl chain component of clay particles inhibited *Staphylococcus aureus* infections [135]. The anti-inflammatory effect of these biopolymer-based medical devices was observed to be due to the inhibition of cytokine expression, including monocyte chemoattractant protein, interleukins, C-reactive protein, tumor necrosis factor, and inducible nitric oxide synthase. The unique features of such medical devices are also linked to higher gene expression, especially those encoding proteins responsible for actively regenerating various tissues, such as intestinal, cardiac, neural, osseous, and vascular tissues. The genes involved in these tissues were cytokeratin, type I collagen, heparan sulfate proteoglycan, prostacyclin, caveolin-1, and thrombomodulin [55,136]. The excellent hemocompatibility of the PHB polymer at the insertion site was reflected by the presence of undetectable lymphocyte levels, indicating poor immune reactions. Thus, PHBV copolymers could be used to develop devices such as coronary stents and patches for the pericardial wall and pulmonary artery [56]. Implants (wound dressings) coated with lysozyme that prevent biofilm formation (via anti-adhesion) have been fabricated from these biopolymers [121,137]. A PHB copolymer fastener coating on implants allows for well-regulated drug release [138,139].

### 3.4. Biocontrol Agents

Recently, PHAs have increasingly been used as biomaterials and anti-infective agents. PHA-based nanocomposites have been developed as bioactive materials that can be used to combat bacterial infection. Some studies have shown that PHAs can be used to control bacterial growth in vivo. However, comprehensive reviews that highlight the role of PHAs in disease prevention and treatment are lacking. Of the few PHA antibiotics currently available, most are against gram-positive bacteria, and only a few can be used against gram-negative bacteria and fungi. Studies have shown that PHA binds and inhibits the function of some microbial cell wall structures, such as the lipid A component of lipopolysaccharides. PHA has also been shown to disrupt bacterial cell membranes and target specific cell wall structures. PHA binding to bacterial membranes caused loss of membrane integrity and bacterial cell death. Hence, PHA can potentially be used as an alternative to current antibiotics. As an alternative to conventional antibiotics, PHAs are relatively safe and can be harmlessly degraded in the environment.

The emergence of drug-resistant bacteria has negatively affected human health, agriculture, and aquaculture. Animal feed supplementation with antibiotics has been banned, and ecologically and economically sustainable biocontrol agents are being investigated [140]. The primary concern is the emergence of resistance among gastrointestinal microflora [141,142]. Modifying PHA functional groups, especially hydroxyl (–OH) and carboxylic acid (–COOH), can result in the production of [(R)-3-hydroxycarboxylic acids, (R)-3HA]. Depolymerization of PHAs to monomers can be performed using the *P. fluorescens* GK13 depolymerase for reducing *S. aureus* infections [143,144,145]. Conjugating these monomers with D-peptides acts as an anticancer agent [122], whereas P3HB/P4HB enhances the angiogenic characteristics of wound and skin healing [126,146]. A variety of hydroxycarboxylic acids, including β-lactones and 2-alkylated 3HB, can be produced by transforming 3HAs for use as oral drugs, such as carbapenem or macrolide antibiotics [99,100,147]. These synthetic molecules possess antimicrobial, antifungal, and antiviral properties. PHB has been used as a growth inhibitor against *Vibrio* spp., *Salmonella* spp., and *Escherichia coli* [148]. Initial studies involved testing these properties against the pathogenic *Vibrio campbellii*. This treatment resulted in a 2-to 3-fold increase in the survival rate of brine shrimp larvae. Indirectly, it proved the potential of PHAs to extend the prospects of providing health benefits to humans [149]. PHAs depolymerization can generate short-chain fatty acids, which are effective antimicrobials against pathogens that cause diseases in giant tiger prawns (*Penaeus monodon*) [150,151].

PHAs as biocontrol agents vary in efficacy depending on their monomeric composition. (R)-3HAs with 8–18C atoms and strong bactericidal activity were obtained from *Streptomyces* sp. JM3 (JN166713). At a minimal inhibitory concentration (MIC) of 1.2–25 mg/mL, it could act against *E. coli* O157:H7, *Salmonella typhimurium* (ATCC 14028), and *Listeria monocytogenes* (ATCC 7644). Chemically modified PHA(3-hydroxy octanoate) was effective as (i) antimicrobial against (a) bacterial pathogens (MIC 2.8–7.0 mM) and (b) the fungi *Microsporum gypseum* and *Candida albicans* (MIC:0.1–6.3 mM) and (ii) (E)-oct-2-enoic and 3-oxo octanoic acid (IC_50_ 1.6–1.7 mM) against lung fibroblasts without affecting mammalian cell proliferation [152]. Biopolymeric films of a nanocomposite nature were produced by combining nanomelanin particles with PHB. This enabled the expression of antibacterial activity against MDR strains of *S. aureus*. It has been proposed for application in medical devices and food materials against microbial infection and oxidation [153].

Pretreating rat synovial fibroblasts (2.5 × 107) infected with *S. typhimurium* (ATCC 14028) (60 µL of 1 × 106 CFU/mL) with 0.8 mg/mL (R)-3HA resulted in higher viability [154]. PHACOS, a modified bacterial polyester, substantially inhibited the biofilm-producing potential of MRSA. This functional modification had a very low inflammatory effect and extremely low levels of cytotoxicity. This helped the adhesion of fibroblasts and considerably reduced the presence of neutrophils and macrophages around PHACOS implants [146]. A unique approach to protect the larvae of giant tiger prawns *P. monodon* against *V. campbellii* was achieved by fortifying the feed with PHB [151]. PHB metabolism led to monomers (3-HB), which, along with its modified form, 3HB methyl ester, has been shown to supply energy in the absence of glucose. This mechanism is expected to be effective during (i) severe brain injuries causing hypoglycemia, (ii) inhibition of reactive oxygen species generation in mice with Alzheimer’s disease (AD), and (iii) inhibition of apoptosis [73]. Based on this information, (D)-BHB, a ketone body, could act as an alternative source of glucose to supply energy. This isomeric form could prevent neuronal death by lowering the concentration of light chain 3 and its associated protein p62. It inhibited the accumulation of autophagosomes, thereby stimulating autophagic flux [155]. The efficiency and production cost of these PHA-based materials can be improved using jatropha oil, vegetable oils, and sugarcane molasses as feed for *Bacillus*, *Cuprividus*, and *Pseudomonas* spp. These modified biopolymeric materials have been used in wound dressing bandages, preventing shrimp mortality and improving food storage [59,121,156,157,158]. A brief presentation of the applications of PHAs as biocontrol agents has been presented in Table 3.

## 4. Perspectives

Biopolymers, such as PHAs, have a high potential for commercialization owing to their unique properties compared to many other biopolymeric substances. In the future, all medical devices could be replaced with PHA and their derivatives. Degradation of biopolymers in biological environments occurs through enzymatic and non-enzymatic hydrolysis. These processes don’t involve thermal oxidation, photolysis or radiolysis [159]. PHA degradation takes place with the help of depolymerizing enzymes: depolymerases, 3HB-oilgomer hydrolases, acetyl-CoA hydrolases, and dehydrogenases [143,145,147,160,161,162]. PHA depolymerase consists of a catalytic domain and a substrate-binding domain. Crystalline PHA binds to the substrate binding domain of the enzyme, whereas the catalytic domain initiates the cleaving of the polymer chain [163]. The degradation process is dependent upon the composition, stereoregularity, additives, crystallinity, and accessibility of the polymer. The end products of the degradation process are either (i) CO_2_ and water under aerobic conditions or (ii) methane, CO_2_, and water under anaerobic conditions [164]. The rate of degradation is affected by various factors: microbial population and diversity, temperature, pH, nutrient supply, moisture level, and properties of the polymer (composition, and crystallinity). The rate of polymer synthesis in *Cupriavidus necator* under N-free medium was 10-fold faster than its degradation [162]. Extracellular degradation is influenced by the length of the side chain of PHA. Higher degradability was observed in PHA with longer side chains [165]. PHA production in *Bacillus* species was observed to peak from 72–120 h, whereas its depolymerization was reported to be from 96–192 h of incubation. The duration of these processes varied with bacterial strain and feed [166]. These devices dissolve over time and are much easier to sterilize than traditional metal or plastic devices. This would be beneficial in cases where repeated surgery is required or where a device must be replaced due to wear and tear. One potential application of this technology is in developing artificial organs that can be implanted into the human body. These devices can also be used in prosthetics to help restore functionality in damaged limbs. PHAs can serve as templates to produce recombinant proteins, which can be used as therapeutic agents for treating various diseases. PHAs are also used to produce bone cement owing to their strong mechanical properties. These are only a few of the many potential biomedical applications of PHA. Novel technologies are being developed that will allow the use of these materials in even more applications and medical research efforts. Despite such promising benefits, few challenges must be addressed before full-scale commercialization [167]. Research on modifying the properties of PHAs must focus on lowering the melting point and glass transition temperature and improving the tensile strength, elastic modulus, and elongation. These features govern the molecular weight, which in turn is influenced by the monomers present in the copolymers [168,169]. To counter the feed cost, biowastes and culture conditions must be evaluated and optimized, genetically modifying the expression of PHA operons and synchronizing the expression of depolymerase genes and PHA biosynthesis with auto-cell lysis [170,171,172,173]. These manipulations are expected to lead to the cost-effective commercial production of PHAs.

## 5. Conclusions

The use of plastic-based materials has reached a stage where they have become indispensable. However, due to their non-biodegradable nature, their applications turn non-ecofriendly and contribute to the financial burden of controlling environmental pollution. Hence, bioplastics, especially co-polymers of PHAs, have provided an opportunity to use them in diverse fields, especially for medical applications. The major limitation of PHAs is their costly commercial-level production. The following two strategies can help overcome this limitation to a large extent. Firstly, using biowastes as feed can lead to a 45% reduction in production costs. Secondly, their usage for high-valued medical applications such as sutures, implants, drug delivery agents and carriers, wound healing meshes, tissue engineering, and biocontrol agents can contribute to their application.

## Figures and Tables

**Table 1:** Diversity of biomedical applications of polyhydroxyalkanoates and their derivatives in tissue engineering.

Bioproducts	Biopolymer Producers	Biomedical Applications	References
PHB	*Bacillus cereus* SPV	Scaffold blend carrying fabricated bacterial cellulose enabled enhancement (12%) in a proliferation of cartilaginous murine ATDC5 cells	[64]
		Scaffolds blended with bioactive glass and sugar had higher (15%) proliferation of MG-63 osteoblast cells with higher biocompatibility	[63]
		PHA blended with bioactive glass and multi-walled carbon nanotubes can be used for bone tissue engineering having electrically conductive sensing features	[63]
	*Cupriavidus eutrophus* B10646	Plasma-treated polymer film improved the adhesive ability of NIH3T3 mice fibroblast	[62]
	*Azotobacter chroococcum* 7B	The blending of PHB with hydroxyapatite and alginate hydrogel embedded with mesenchymal stem cells enhanced (3.5 times) the regeneration of critical bone defects in rats	[31]
	*Isoptericola variabilis* PPLAT 012	A blend of biofilm with polylactate did not have any toxicity toward mouse fibroblast L929 cells	[67]
	*Paraburkholderia xenovorans* LB400	Microfibers with high cytocompatibility withBalb/3T3 fibroblast	[77]
	*Escherichia coli*	Composites containing zirconium dioxide and Herafill allowed the formation of bone tissue at the implant site in the rat femora	[37]
	Commercial	PHB-gelatin electrospun scaffold was effective for regenerating skin in diabetic wounds. It achieved wound healing at an enhanced rate with higher sweat glands and hair follicles	[34]
Poly(3-hydroxyoctanoate)	*Pseudomonas mendocina* CH50	PHA composite of bioactive glass component had a higher regeneration rate for human keratinocytes HaCaT	[57]
		Composite with cardiac patch having vascular growth factor exhibited 2.5-fold higher C2C12 myoblast cell proliferation	[28]
Poly(3-hydroxybutyrate-co-3-hydroxyvalerate) [P(3HB-3HV)]	*Bacillus* sp. WW	Scaffolds enabled attachment and growth of proliferative cells	[66]
	*Pseudomonas putida* CA3	PHA fibers blended with polycaprolactone resulted in enhanced durability (2.3-times) and growth (3.8-times) of human-induced pluripotent stem cells	[75]
	Commercial	PHA copolymer membrane in cerium oxide nanoparticles healed diabetic wounds through a higher level of vascularization and proliferation of cells	[29]
		A well-regulated myofibroblast formation resulted in restricting the unnecessary scar formation in a mouse wound	[30]
		Nanofibers of high molecular Keratin with nanoparticles had improved wound healing properties and antimicrobial properties observed in a rat wound healing model	[32]
		PHA blended with GelMA/epidermal growth factor helped in faster wound healing which proved suitable for angiogenesis and conducive cellular response in diabetic wound	[33]
P(3HB-3HV) [(16 mol% HV (3-hydroxyvalerate)	*Alkaliphilus oremlandii* OhiLAs	Curcumin-modified polyaniline/PHA as electrically conductive scaffolds Composite (PHA/polyaniline and curcumin) has the ability to enhance the proliferation of fibroblast cells. Also used for repairing damaged tissues	[59]
P(3HB-3HO) (1.0:25)	*B. cereus* SPV and *P. mendocina*	Nanofiber scaffolds provide a conducive environment for effective cartilage repair. Provide long-term mechanical stability and durability. Support the self-healing potential of the body	[51]
P(3HB-4HB-3HV)	*Cupriavidus necator* DSM 545	PHA electrospun fabrication of meshes for the growth of stem cells	[48]
P(3HB-3HV-3HHx)	*Aeromonas hydrophila 4AK4* (recombinant)	Scaffolds loaded with MSCs (from the umbilical cord) improved and looked like normal liver	[49]
Medium chain length PHA (mcl-PHA)	*Pseudomonas chlororaphis* DSM 19603	Polymer film had strong adhesability to human skin with no side effects	[76]

**Table 2:** Diversity of biomedical applications of polyhydroxyalkanoates and their derivatives in drug carriers and delivery.

Bioproducts	Biopolymer Producers	Biomedical Applications	References
PHB	*B. cereus* VIT- SSR1	PHA-chitosan with curcumin enabled cell viability of up to 98% in mouse fibroblasts	[119]
	Commercial	Porous 3D implants enhanced the rate of regeneration of cranial defects in rats. Suitable for reconstructive osteogenesis	[56]
PHA	*B. cereus* SPV	PHA loaded with Docetaxel killed cancerous cells (U-87MG)	[125]
P(3HB-3HV)	*B. cereus* FA11	PHA-PEG with Epirubicin inhibited pathogens: *Escherichia coli* ATCC 11775, *Pseudomonas aeruginosa* ATCC 27853, and *Staphylococcus aureus*	[120]
	*C. necator* H16	PHA-PVA with derivatives of porphine used for photodynamic treatment of cancerous cells (Killed 94% of colon cancer cells)	[98]
P(3HB-3HV) (6.5 mol% HV)	Hydrogen-oxidizing organisms	PHA-Doxorubicin killed HeLa cells	[118]
P(3HB-3HV) (12 mol% HV)	*B. cereus* FA11	PHA-PVA loaded with Ellipticine inhibited 65% of cancerous cells	[123]
P(3HB-3HV) (16 mol% HV)	*A. oremlandii* OhiLAs	PHA-polyaniline with curcumin caused the arrest of the cell cycle at G0/G1 phase leading to death	[59]
P(3HB-3HO) (10 mol% HO)	*Sinorhizobium fredii*	PHA-PEG-Folic acid with Doxorubicin kills 3-fold higher HeLa cells	[97]

**Table 3:** Diversity of biomedical applications of polyhydroxyalkanoates and their derivatives as biocontrol agents.

Bioproducts	Biopolymer Producers	Biomedical Applications	References
PHB	*Bacillus mycoides* DFC1	Film coated with vanillin was useful for food storage. As it could inhibit the growth of *S. aureus* MTCC737, *Shigella flexneri*, and *Salmonella typhimurium* at MIC: 80 μg/g of film, whereas for fungal genera *Penicillium* and *Aspergillus*, a MIC was 100 μg/g	[158]
	*Brevibacterium casei* MSI04	Biopolymer film blended with nanomelanin could completely inhibit *S. aureus* biofilm	[153]
P(3HB-3HV) (16 mol% HV)	*A. oremlandii* OhiLAs	Curcumin-carrying scaffold was effective as a biocontrol agent against diverse pathogens: *S. aureus*, *Bacillus subtilis*, *P. aeruginosa*, and *E. coli* XL1B	[59]
P(3HB-3HV)(25 mol% HV)	*Ralstonia eutropha* H16	PHA copolymer nanofiber loaded with biocide wound dressing inhibited 98.9% of *P. aeruginosa* infection	[157]
P(3HB-3HHx) (17%mol HHx)	*C. necator* Re2001/pCB81	A film with lysozyme could inhibit (9%) *Rhodococcus opacus* biofilm	[121]
P(3HB-3HO-3HD)	*Streptomyces* sp. JM3	Modified PHAs as 3 hydroxy acids effective in inhibiting infections caused in fibroblasts by pathogens: *Listeria monocytogenes*, *E. coli* O157:H7, *S. typhimurium*	[154]
P(3HO-3HD) (25.6:74.4)	*P. mendocina* CH50	Biofilm loaded with lime oil as a biocontrol agent against *E. coli* and *S. aureus* infections	[156]

## Data Availability

Not applicable.
